# Effect of Biosynthesized Gold and Silver Nanoparticles Using *Alchemilla vulgaris* Extract and Their Synergistic Action with Subinhibitory Concentrations of Ampicillin Against Staphylococci

**DOI:** 10.3390/antibiotics15030250

**Published:** 2026-02-27

**Authors:** Ján Király, Gabriela Gregová, Patrícia Hudecová, Vanda Hajdučková, Simona Hisirová, Nikola Dančová, Peter Takáč, Valéria Verebová, Zdenka Bedlovičová

**Affiliations:** 1 Department of Microbiology and Immunology, The University of Veterinary Medicine and Pharmacy in Košice, 041 81 Košice, Slovakia; jan.kiraly@uvlf.sk (J.K.); patricia.hudecova@student.uvlf.sk (P.H.); vanda.hajduckova@uvlf.sk (V.H.); simona.hisirova@student.uvlf.sk (S.H.); 2 Department of Public Veterinary Medicine and Animal Welfare, The University of Veterinary Medicine and Pharmacy in Košice, 041 81 Košice, Slovakia; gabriela.gregova@uvlf.sk (G.G.); nikola.dancova@uvlf.sk (N.D.); 3 Department of Pharmacology and Toxicology, The University of Veterinary Medicine and Pharmacy, Komenského 73, 041 81 Košice, Slovakia; peter.takac@uvlf.sk; 4 Department of Chemistry, Biochemistry and Biophysics, The University of Veterinary Medicine and Pharmacy in Košice, 041 81 Košice, Slovakia; valeria.verebova@uvlf.sk

**Keywords:** nanoparticles, gold, silver, green synthesis, *Alchemilla vulgaris*, ampicillin, subinhibition concentrations, synergism, multiresistance, biofilm, *Staphylococcus* spp.

## Abstract

Background: Staphylococci, recognized for their virulence and antibiotic resistance, are important in both human and veterinary medicine. Loss of sensitivity to beta-lactam antibiotics, such as ampicillin, complicates therapy, prompting the search for alternative antibacterials or ways to increase drug efficacy. Silver and gold nanoparticles (AgNPs, AuNPs) are promising on their own or in combination with antibiotics. Methods: The aim of this study is to compare the biological activity of pure, washed AgNPs and AuNPs with biosynthesized nanoparticles from Alchemilla vulgaris (AgNPs-Av and AuNPs-Av). Their antibacterial, antibiofilm, and biofilm-eradication effects on the tested antibiotic-resistant, biofilm-forming staphylococci (Methicillin-resistant *Staphylococcus aureus* (MRSA) and multiresistant Non-*aureus* staphylococci and mammaliicocci (NASM)) were evaluated using in vitro microdilution methods. Results: AgNPs-Av and AuNPs-Av inhibited bacterial growth at 50 μg/mL, while a significant suppression of biofilm formation was observed at just 25 μg/mL. Our research showed that neither AuNPs-Av nor AuNPs disrupts bacterial biofilm. AgNPs-Av effectively eradicated the biofilm at 50 μg/mL. NPs and ampicillin at subinhibitory antibiotic concentrations against the tested staphylococci. The results showed significant antibacterial and antibiofilm effects (*p* = 0.001). Partially, biofilm-eradication activity and strong antibiotic potentiation were also detected. Conclusions: These findings highlight the importance of rational combination therapy to improve antibiotic effectiveness and reduce bacterial resistance.

## 1. Introduction

The increasing antimicrobial resistance of bacterial pathogens raises concerns about the effectiveness of therapeutic approaches aimed at eradicating them from the host organism and thus treating the diseases they cause. During a long period when antibiotics saved many lives of people and animals, their widespread use has led to an enormous increase in resistant bacteria. Bacterial infections are once again a threat to public health, livestock, and food security. The ATB resistance crisis has arisen as a result of the overuse and misuse of these drugs, as well as the insufficient development of new pharmaceutical drugs. It is expected that by 2050, deaths due to ATB resistance may increase to 10 million cases per year, which would exceed the number of deaths from cancer [1,2].

From the perspectives of human and veterinary medicine, attention is primarily given to multiresistant (resistance to 3–6 antibiotics of different types) and polyresistant (resistance to 6 or more antibiotics of different types) strains. Bacteria have developed numerous mechanisms of resistance to antimicrobial agents over time. The most common methods include altering the target bacterial structure, producing enzymes that modify or degrade ATB, reducing bacterial membrane permeability, and increasing efflux pump expression. A critical and effective method of resistance is the formation of a biofilm, in which an exopolysaccharide glycocalyx protects bacterial cells [3,4].

Staphylococci are a genus of bacteria with over 50 species, many of which are part of the normal microbiota in humans and animals [5]. They include *S. aureus*, Non-*aureus* staphylococci and mammaliicocci (NASM), which can develop antibiotic resistance, making treatment difficult [6]. To ensure sustainable livestock health, strict hygiene standards must be maintained. However, infectious diseases that affect the limbs, skin, or mammary glands are common in cattle farming. This is caused by regular contact with the environment and humans [7,8]. Although the cause of such diseases involves many external factors and the presence of pathogenic microorganisms (bacteria, fungi, viruses), species of the genus *Staphylococcus*, such as *S. aureus*, *S. hyicus*, *S. chromogenes*, *S. simulans*, *S. equorum*, *S. xylosus*, *S. pseudintermedius*, and *Mammaliicoccus sciuri* (formerly *S. sciuri*), play a significant role in livestock health risks [9,10,11,12,13,14,15,16].

Resistance and biofilm formation are important because they decrease the effectiveness of treatments for bacterial infections. They also motivate the search for alternative therapies. Nanotechnology, particularly nanoparticles (NPs), is emerging as a promising approach to fight bacterial infections. This method enables the creation of various types of antimicrobial nanomaterials, which can be tailored to target specific bacteria, penetrate biofilms, and reduce the risk of resistance. It also offers high flexibility and multifunctionality [17]. The metal NPs of silver (Ag), copper (Cu), selenium (Se), nickel (Ni), gold (Au), zinc oxide (ZnO), titanium dioxide (TiO_2_), and iron oxide (Fe_3_O_4_) attract the most attention because of their unique antimicrobial properties [18]. The size, surface area, morphology, net charge, and physicochemical properties of NPs are important parameters that influence their antimicrobial effects through different mechanisms [19]. As NP size decreases, the surface area-to-volume ratio increases, and their large surface areas enable better interactions with microorganisms, thereby significantly affecting their antimicrobial effects. For metal NPs, it has been reported that positively charged NPs bind more tightly to negatively charged bacterial surfaces and exhibit stronger antimicrobial effects [20]. It has also been shown that spherical NPs have a stronger antimicrobial effect because, due to their larger surface area, they release more ions [21]. The antibacterial effect is also affected by the type of capping agent used, as well as the pH and ionic strength of the medium. Capping agents adsorbed on the surface of NPs serve as stabilizers, preventing mutual aggregation. These agents impact the biological activities and the structural and physicochemical properties of NPs [20]. Plant extract coatings on NPs act as reducing agents during their synthesis. They also provide a coating that prevents aggregation and significantly improves their antibacterial efficacy [22]. Silver NPs (AgNPs) are among the most widely used NPs and demonstrate potent antibacterial and antibiofilm effects by disrupting the integrity of bacterial membranes. Exposure of cells to AgNPs leads to the production of reactive oxygen species that interact with bacterial DNA, lipids, and proteins, ultimately leading to bacterial cell death [23,24]. The antibiofilm activity of AgNPs works by preventing bacterial adhesion to surfaces, either by disrupting intermolecular forces or by inhibiting quorum-sensing systems [20]. The benefit of using NPs is their synergistic effect with antibiotics. Their combination can lower the required antibiotic concentration and reduce their toxicity to the organism [25,26]. NPs target bacteria by disrupting cell membranes, generating reactive oxygen species, and interfering with cellular processes. These multiple mechanisms render bacteria ineffective, reducing the likelihood of resistance development. Therefore, resistance through mutation is unlikely, and using NPs with antibiotics is a promising way to prevent bacterial resistance [27]. Using antibiotics with NPs effectively inhibits both Gram-positive bacteria, such as *S. aureus*, and Gram-negative bacteria, including *E. coli* and *P. aeruginosa*, even multidrug-resistant strains [28]. The synergistic effect of AgNPs with antibiotics is effective not only in their antibacterial activity but also in preventing the formation and spread of persistent bacterial biofilms [29,30].

Gold NPs (AuNPs) are also attracting interest among the scientific community. Although they have received less attention than AgNPs, many studies have already demonstrated their potential biological activity [31,32,33]. There are several reasons why they are appealing, including their ease of surface modification, low cytotoxicity, high surface-to-volume ratio, and excellent biological compatibility. These properties confer AuNPs with effective antibacterial activity, aiding bacterial degradation and cell death [34]. Published high-quality analyses describe their antibacterial activity by disrupting the cell membrane, reducing metabolism, and interfering with bacterial transcription. AuNPs can adhere to the bacterial membrane and penetrate the cytoplasm, inhibiting ATPase production and the ribosomal subunit responsible for tRNA protection, leading to disrupted metabolism and transcription [35]. Previous studies have indicated that combining drugs with AuNPs produces stronger, longer-lasting antibacterial effects, emphasizing the potentiation effect of these combinations [33].

This study aims to examine the effect of AgNPs and AuNPs biosynthesized using *Alchemilla vulgaris* (or washed NPs) on biofilm-forming, multidrug-resistant staphylococcal strains. In particular, their antibacterial, antibiofilm, and biofilm-eradication effects on the tested staphylococci were evaluated.

An innovative aspect of our study, which was missing in previous research, is the investigation of the synergistic effect achieved by combining biologically active NPs with subinhibitory concentrations of the antibiotic ampicillin against these bacteria.

## 2. Results

For this study, biosynthesized NPs were prepared using an extract from the medicinal plant *Alchemilla vulgaris*. The aim was to improve the biological effects of these particles. Published research and initial experiments directed the choice of silver and gold NPs. The biological activity of these biosynthesized NPs (AgNPs-Av and AuNPs-Av) was compared to that of pure, washed NPs (AgNPs and AuNPs). The antibacterial, antibiofilm, and biofilm-eradication effects of silver and gold were analyzed. To evaluate the synergistic effect of NPs combined with antibiotics, ampicillin was used. Minimum inhibitory concentrations (MICs) of ATB were determined for each tested strain (Table A1).

### 2.1. Antibacterial Effect of AgNPs, AuNPs, and AgNPs-Av, AuNPs-Av

**Figure 1:**
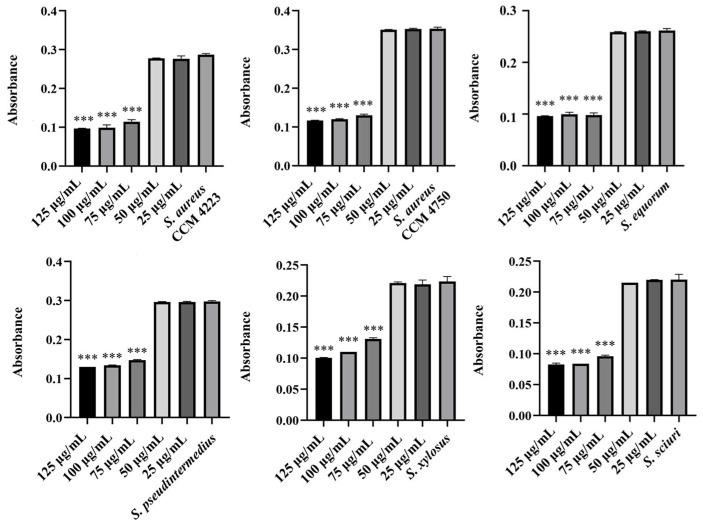
Antibacterial effect of AgNPs against tested staphylococci. Description: *** significant production of biofilm *p* < 0.001.

**Figure 2:**
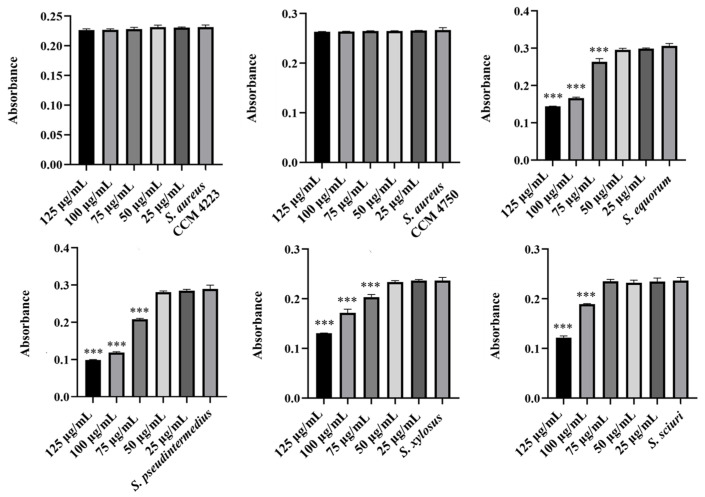
Antibacterial effect of AuNPs against tested staphylococci. Description: *** significant production of biofilm *p* < 0.001.

The observed antibacterial effects of pure washed AgNPs and AuNPs against selected strains of staphylococci were the same only against NASM, while a slightly increased inhibition was recorded with AgNPs. Additionally, they inhibited *S. aureus* strains, while AuNPs had no effect. The lowest concentration of pure NPs required to inhibit bacterial growth was 75 mg/mL (Figure 1 and Figure 2).

**Figure 3:**
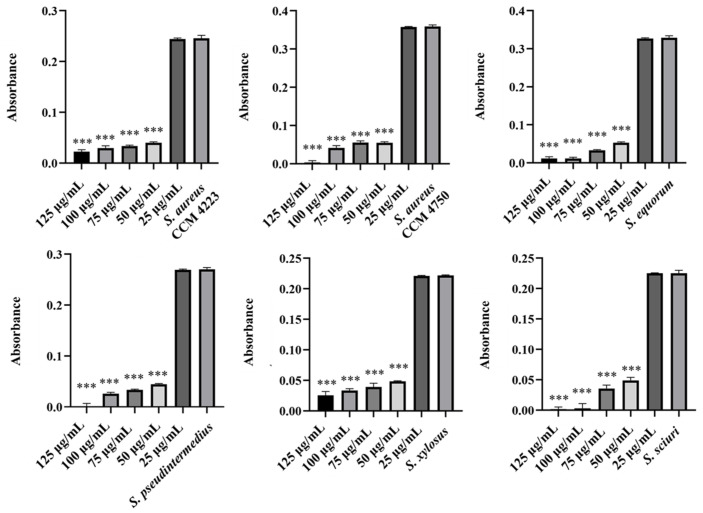
Antibacterial effect of AgNPs-Av against staphylococci. Description: *** significant production of biofilm *p* < 0.001.

**Figure 4:**
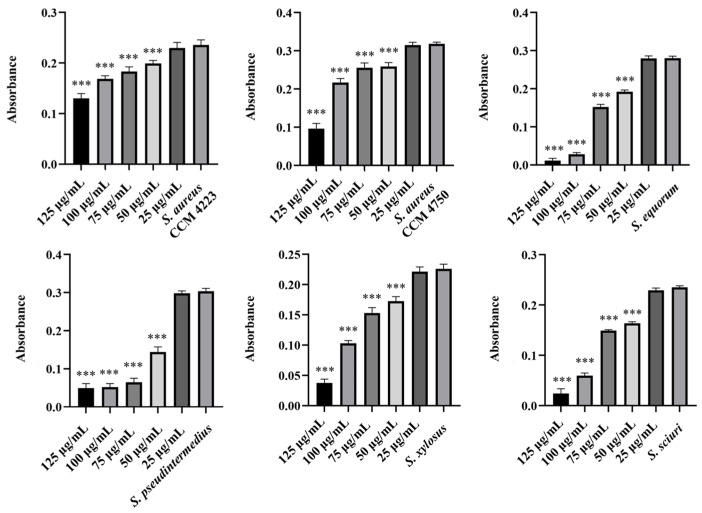
Antibacterial effect of AuNPs-Av against staphylococci. Description: *** significant production of biofilm *p* < 0.001.

A significant inhibitory effect on bacterial growth was observed with AgNPs-Av and AuNPs-Av. They exhibited a strong antibacterial effect against both SM strains and *S. aureus* strains. The graphs demonstrate higher inhibitory activity for AgNPs (Figure 3), compared to AuNPs (Figure 4), with both showing the same significance level at 50 μg/mL.

### 2.2. Antibiofilm Effect of AgNPs, AuNPs and AgNPs-Av, AuNPs-Av

**Figure 5:**
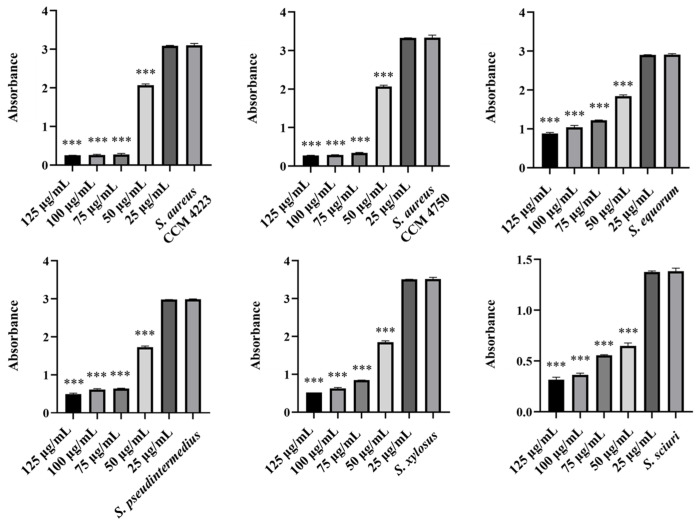
Antibiofilm effect of AgNPs against staphylococci. Description: *** significant production of biofilm *p* < 0.001.

**Figure 6:**
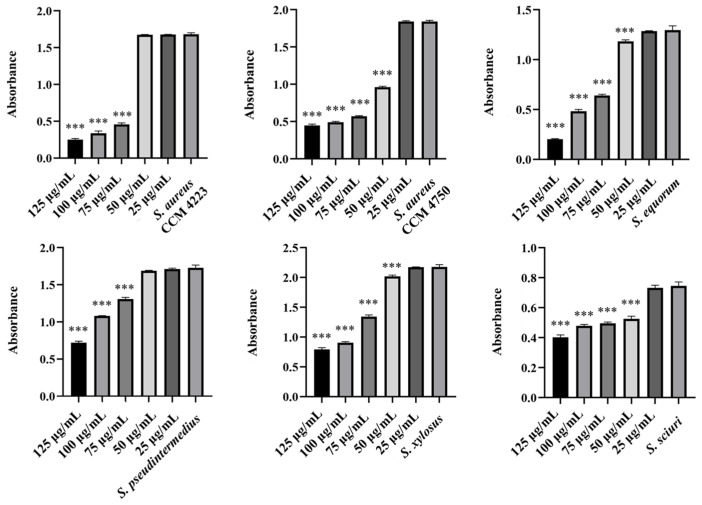
Antibiofilm effect of AuNPs against staphylococci. Description: *** significant production of biofilm *p* < 0.001.

Monitoring the effects of pure AgNPs and AuNPs on biofilm formation revealed significant antibiofilm activity against all tested strains (*S. aureus* and NASM) for both metals. The results highlighted the impact of silver on biofilm formation (Figure 5 and Figure 6).

**Figure 7:**
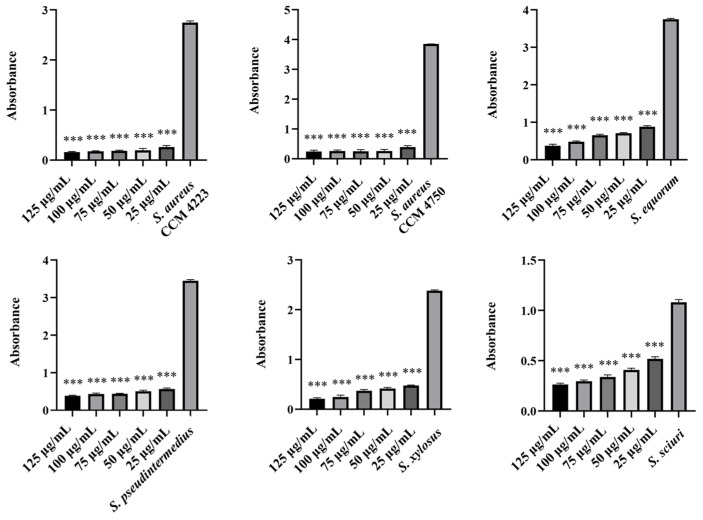
Antibiofilm effect of AgNPs-Av against staphylococci. Description: *** significant production of biofilm *p* < 0.001.

**Figure 8:**
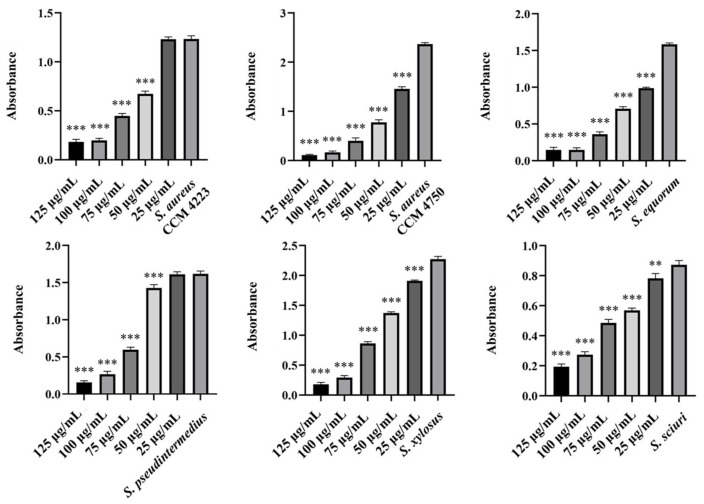
Antibiofilm effect of AuNPs-Av against staphylococci. Description: ** significant production of biofilm *p* < 0.01; *** significant production of biofilm *p* < 0.001.

A notable antibiofilm effect was observed at a concentration of 50 μg/mL. Significant results were achieved with AgNP-Av and AuNPs-Av. Measurements of biofilm formation indicated that AgNP-Av had a greater effect than gold, even though both metals had the same inhibitory concentration against biofilm, 25 μg/mL (Figure 7 and Figure 8).

### 2.3. Biofilm-Eradicating Effect of AgNPs, AuNPs, and AgNPs-Av, AuNPs-Av

**Figure 9:**
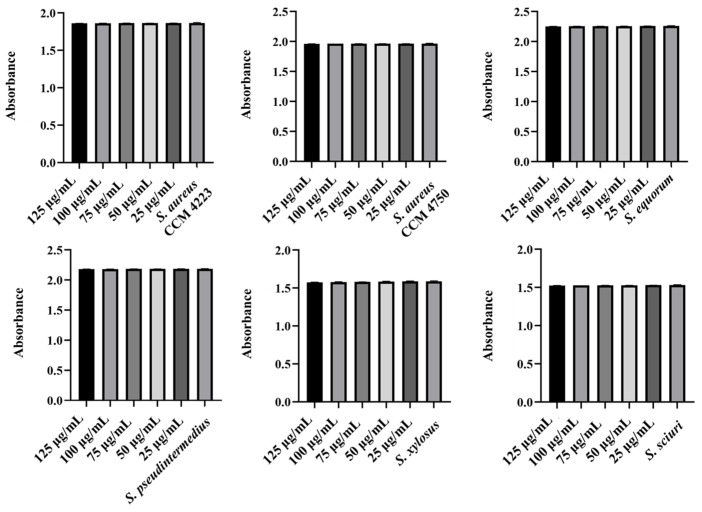
Biofilm-eradication effect of AgNPs against staphylococci.

**Figure 10:**
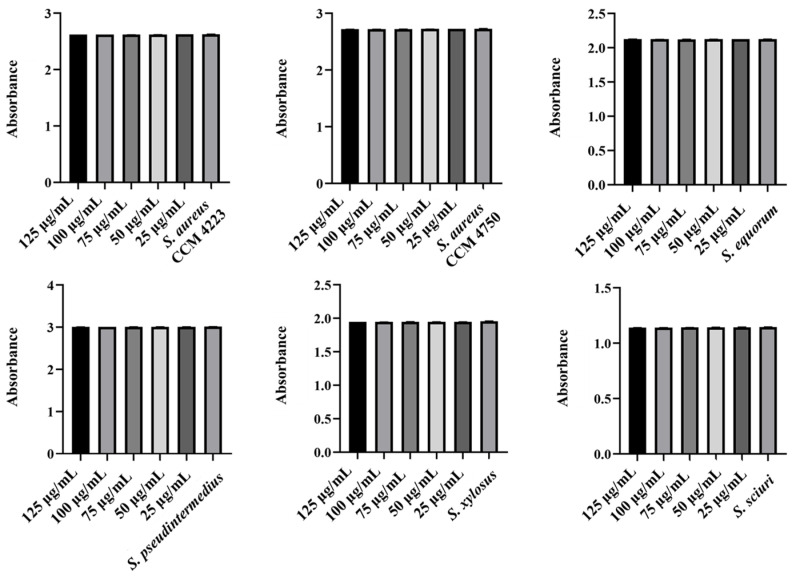
Biofilm-eradication effect of AuNPs against staphylococci.

The experiments in this study aimed to observe the effects of individual NPs. We assessed their ability to disrupt biofilms formed by selected staphylococcal strains. Additionally, we identified the lowest concentration required to eliminate the biofilm. Measurements of the eradication activity of pure AgNPs and AuNPs did not demonstrate their ability to disrupt biofilms (Figure 9 and Figure 10). Notably, we did not observe this ability, even with AgNPs, which previous experiments indicated have higher biological activity.

**Figure 11:**
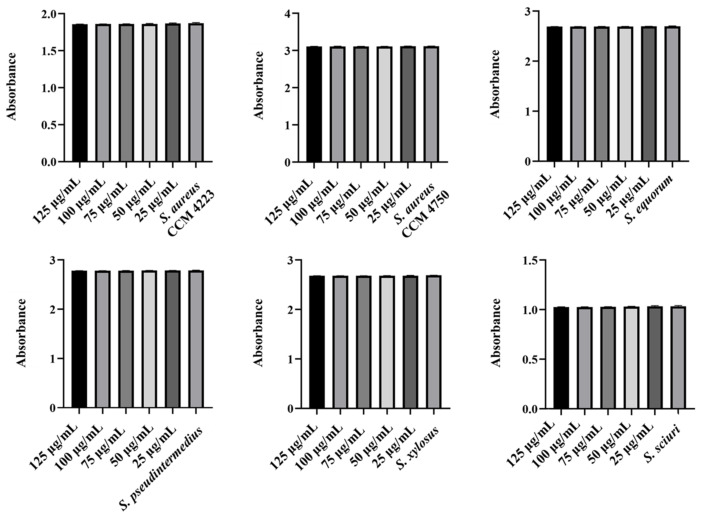
Biofilm-eradication effect of AuNPs-Av against staphylococci.

**Figure 12:**
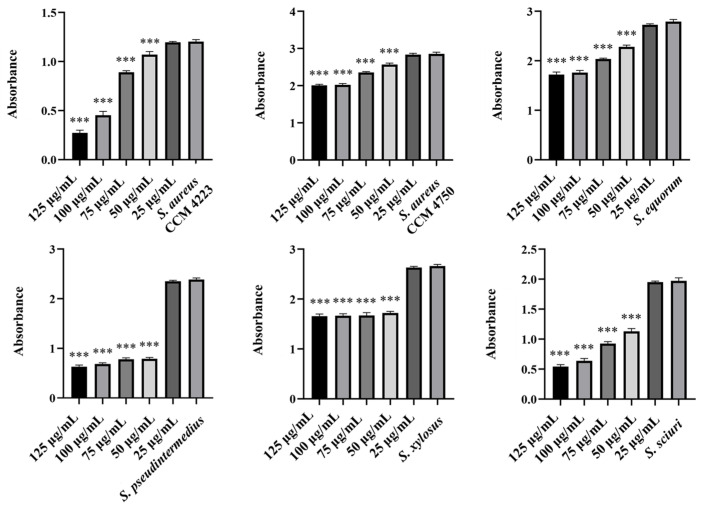
Biofilm-eradication effect of AgNPs-Av against staphylococci. Description: *** significant production of biofilm *p* < 0.001.

We finally assessed the eradication effects of biosynthesized AgNPs-Av and AuNPs-Av. For AuNPs-Av, the results were consistent with those of pure AuNPs; there was no effect on biofilm disruption (Figure 11). In contrast, AgNPs-Av showed more promising results, with a significant effect on biofilm eradication. Notably, the minimum active concentration remained at 50 μg/mL, consistent with the antibacterial activity measurement for AgNPs-Av (Figure 12).

The significance of the lowest effective concentrations of the evaluated NPs (washed NPs, biosynthesized NPs) in the tested staphylococcal isolates is summarized in Table A2 (Appendix A).

### 2.4. Synergistic Action of Biosynthesized Nanoparticles with Subinhibitory Concentrations of Antibiotics

Further experiments aimed to observe the combined synergistic effect of selected subinhibitory concentrations of biosynthesized NPs. These concentrations were chosen based on previous measurements. The experiments used subinhibitory concentrations of the antibiotic for each bacterial strain tested.

**Figure 13:**
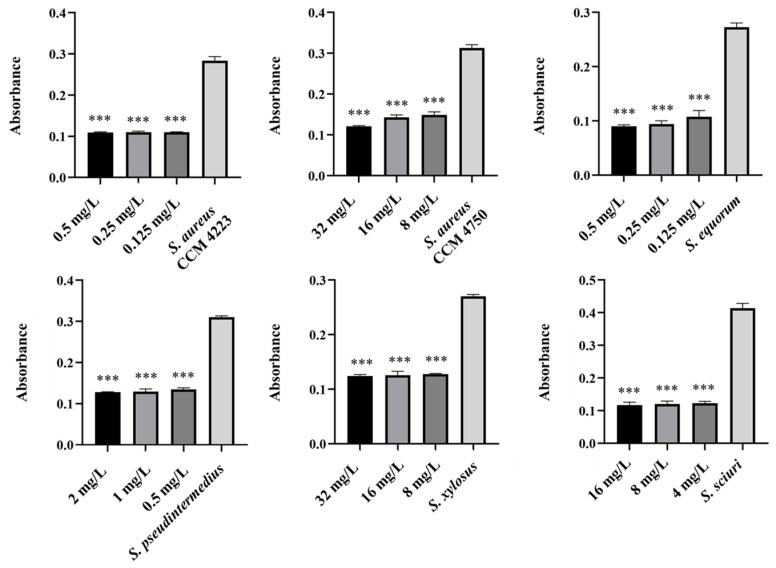
Antibacterial activity of synergistically acting AgNPs-Av with subinhibitory concentrations of ampicillin against staphylococci. Description: *** significant production of biofilm *p* < 0.001.

**Figure 14:**
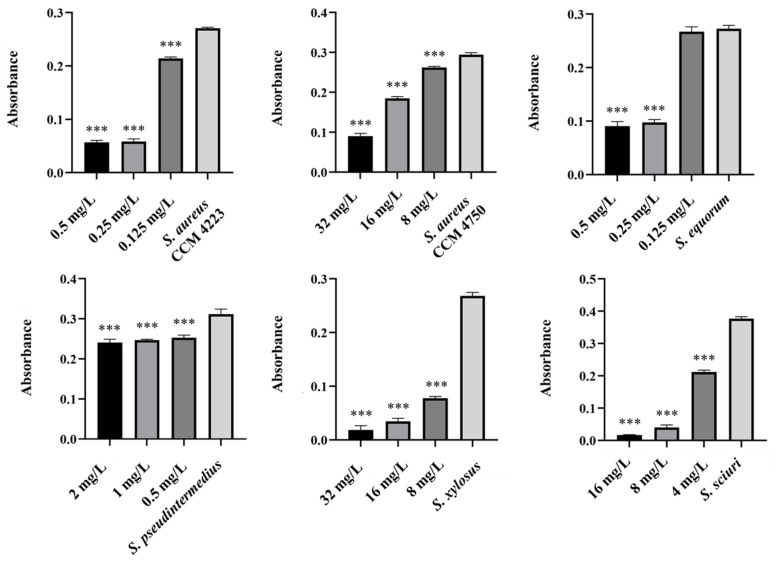
Antibacterial activity of synergistically acting AuNPs-Av with subinhibitory concentrations of ampicillin against staphylococci. Description: *** significant production of biofilm *p* < 0.001.

To test the synergistic effect of NPs and antibiotics on bacterial growth inhibition, NPs were used at half the concentration where a significant antibacterial effect was still observed. Although AuNPs-Av was less effective (75 μg/mL), both AuNPs-Av and antibiotics were used at the same concentration (25 μg/mL) to evaluate AgNPs-Av synergism. This approach maintained consistent conditions, including concentration, as a basis for future application studies. The tested antibiotics corresponded with the MIC values determined for each bacterial strain and its two-fold dilutions. The data indicate that combining subinhibitory concentrations of NP and ATB inhibits bacterial growth even at doses where each agent alone was minimally effective or ineffective. AgNPs-Av (25 μg/mL) demonstrated strong antibacterial activity at all subinhibitory antibiotic concentrations across all strains (Figure 13). Notably, AuNPs-Av (25 μg/mL) with subinhibitory ATB also demonstrated significant inhibitory activity across all tested strains, similar to the results with AgNPs-Av (Figure 14).

**Figure 15:**
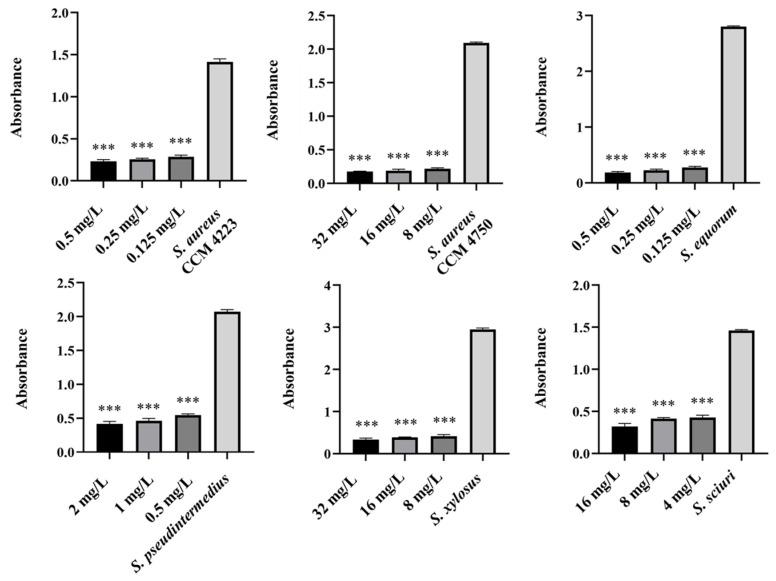
Antibiofilm activity of synergistically acting AgNPs-Av with subinhibitory concentrations of ampicillin against staphylococci. Description: *** significant production of biofilm *p* < 0.001.

**Figure 16:**
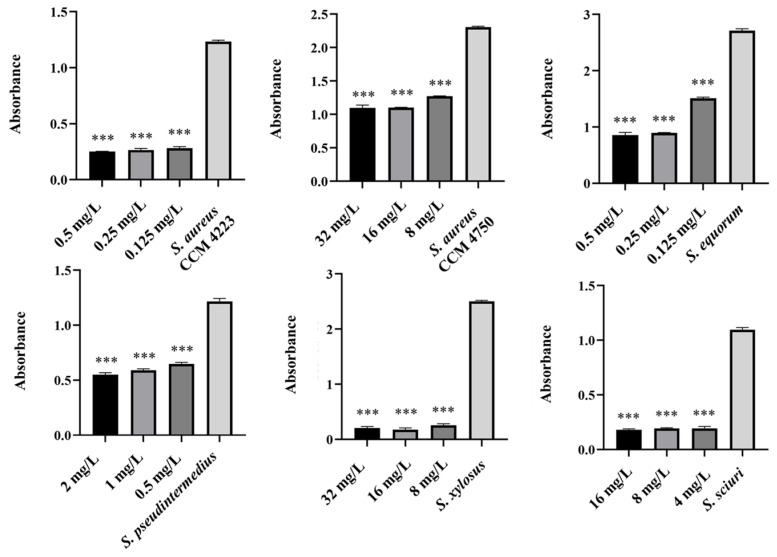
Antibiofilm activity of synergistically acting AuNPs-Av with subinhibitory concentrations of ampicillin against staphylococci. Description: *** significant production of biofilm *p* < 0.001.

Biofilm inhibition was assessed using the same method as for evaluating the synergistic antibacterial effect of NPs and antibiotics. In these experiments, the NPs concentration decreased because, in previous tests, their antibiofilm effect was significantly higher even at 25 μg/mL. Therefore, 12.5 μg/mL was chosen as the subinhibitory concentration for biofilm formation, which is half the lowest concentration that showed significant effects when NPs were tested alone. The same concentration was used to evaluate the effects of AgNPs-Av and AuNPs-Av, ensuring consistency for future studies. Results show a strong synergistic effect of NPs and antibiotics at all tested concentrations across all strains, not only for AgNPs-Av (Figure 15) but also for AuNPs-Av (Figure 16). Additionally, the graphs indicate that silver NPs exhibit stronger activity.

**Figure 17:**
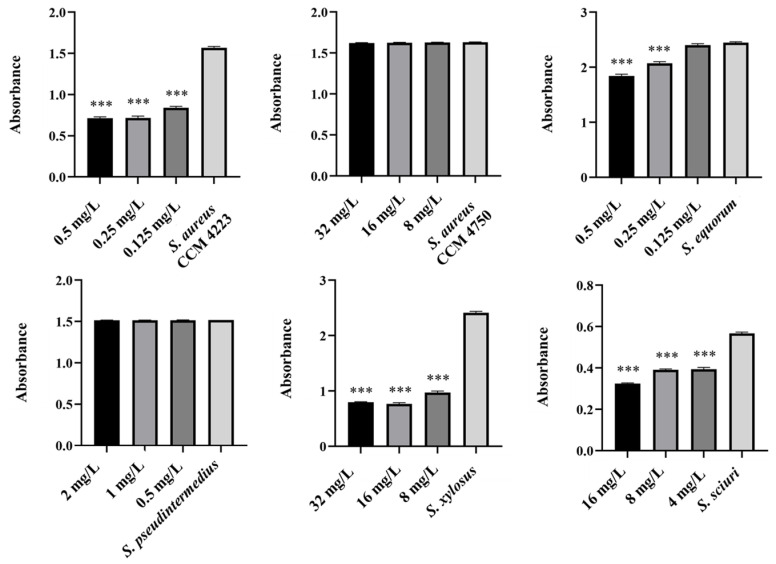
Biofilm-eradication activity of synergistically acting AgNPs-Av with subinhibitory concentrations of ampicillin against staphylococci. Description: *** significant production of biofilm *p* < 0.001.

**Figure 18:**
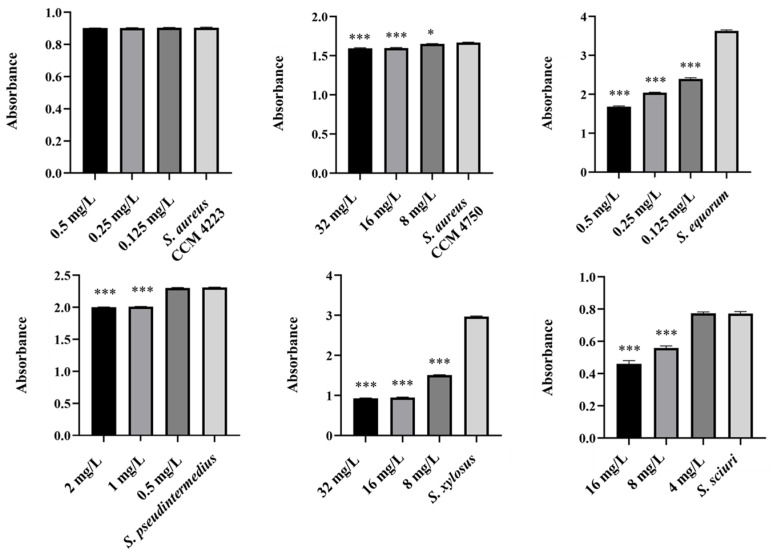
Biofilm-eradication activity of synergistically acting AuNPs-Av with subinhibitory concentrations of ampicillin against staphylococci. Description: * significant production of biofilm *p* < 0.05; *** significant production of biofilm *p* < 0.001.

Finally, focus shifted to eradicating biofilm using NPs with ATB. The selected subinhibitory concentration (25 μg/mL) is based on testing the effectiveness of biosynthesized NPs alone, which showed eradication at 50 μg/mL. Under the same conditions and concentrations, the effectiveness of eradication varied with different methods. AgNPs-Av, combined with all tested ATB concentrations, reduced biofilm formation in the biofilm-forming strains (*S. aureus* CCM 4223, *S. xylosus*, *S. sciuri)*. In *S. equorum*, a significant decrease was observed compared to the control at two concentrations of ATB (0.5 and 0.25 mg/L). In MRSA (CCM 4750) and *S. pseudintermedius*, no decrease was observed at any subinhibitory concentration of ATB (Figure 17). The results obtained using AuNPs-Av in combination with ATB (Figure 18) are notable. AuNPs-Av influenced the reduction of biofilm formation in biofilm-forming strain *S. aureus* (CCM 4223) and all NASM strains. Using all subinhibitory concentrations of ATB, significant reductions were observed in *S. equorum* and *S. xylosus*. The measured biofilm-forming values were obtained at two points: *S. pseudintermedius* (2 and 1 mg/L) and *S. sciuri* (16 and 8 mg/L).

### 2.5. Ag and Au Nanoparticles Biosynthesis and Caracterization

The reduction of Ag(+)and Au(3+) ions to their neutral forms, Ag(0) and Au(0), was achieved using ethanolic plant extracts (prepared at room temperature) of *Alchemilla vulgaris*, which served as the reducing, stabilizing, and capping agent [36,37,38]. The observed visual color change was the first detection of the reduction of silver and gold ions into AgNPs and AuNPs. The color of the produced nanosuspensions was red-brown for AgNPs and violet for AuNPs.

**Figure 19:**
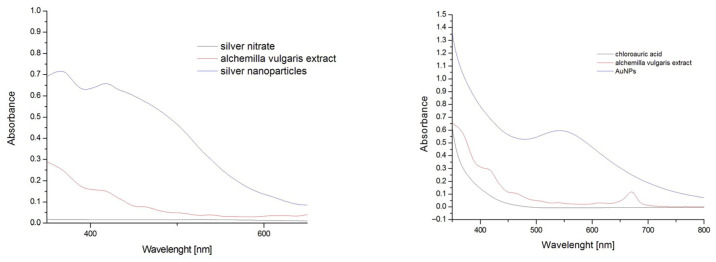
SPR bands occurred during the biosynthesis of silver (**left**) and gold (**right**) NPs.

To monitor the reaction process, the UV-Vis spectra were measured in the region of 350–750 nm each minute directly in the cuvette at a temperature of 80 °C (Figure 19). During the reaction, an increase in absorbance maximum was detected due to the surface plasmon resonance (SPR) phenomenon. The maximum absorption bands were between 415 nm for AgNPs and 537 nm for AuNPs. The reaction process was stopped after reaching the maximum absorbance.

**Figure 20:**
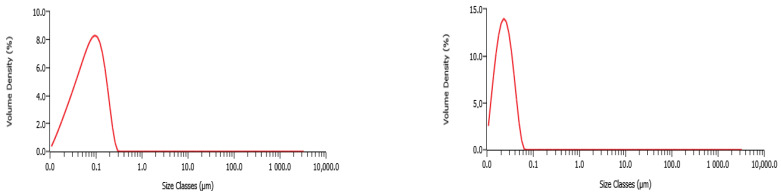
Size distribution measurements of the biosynthesized silver (**left**) and gold (**right**) nanoparticles.

To determine the size and shape of the prepared Au and AgNPs, the nanosuspensions were characterized by size distribution and a microscopy study. By grain size, we mean the size of nanoparticle aggregations (individual NPs joined together via the organic matrix), not the diameter of individual NPs [39]. The grain size distribution data are shown in Figure 20 for both prepared colloidal solutions.

AgNPs-Av were prepared using *A. vulgaris* leaves extract at 80 °C, showing the average grain size distribution between 71.9 nm (Figure 20, left). AuNPs-Av showed a 23.1 nm size distribution of the grains (Figure 20, right).

**Figure 21:**
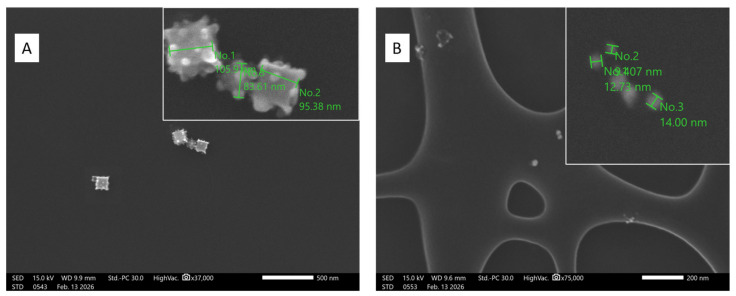
FE-SEM measurements of the biosynthesized of silver (**A**) and gold (**B**) NPs.

Field-emission scanning electron microscopy (FE-SEM) of nanosuspensions revealed the size and morphology of the prepared NPs. The results showed that silver NPs exhibited cubic morphologies and reached approximately 100 nm in length (Figure 21A). The golden NPs were roughly spherical in shape, measuring up to 14 nm (Figure 21B). The observed NP morphology is consistent with other studies that use plant extracts as reducing agents [33,36].

## 3. Discussion

The growing resistance of bacterial pathogens to antibiotics is becoming a serious threat to effective treatment. Administering effective antibiotics in serious bacterial infections is a crucial factor in lowering morbidity and mortality. Therefore, collecting relevant antibiograms should come before empirical antibiotic use [40]. Drugs are distributed throughout people’s tissues and are metabolized at various rates. The consequence of administering an ineffective antibiotic or a subinhibitory concentration that modifies gene expression and, as a result, affects the bacteria’s physiology [41,42]. Another critical factor that enhances bacterial virulence and the emergence of resistance is the bacteria’s ability to form biofilms, common in *S. aureus* and NASM [25,43].

Many studies highlight the potential of using NPs, such as silver and gold. Currently, the properties of AgNPs are well documented, as they have attracted significant attention due to their antibacterial effects, low toxicity, and numerous applications in vitro and in vivo [44].

Washed AgNPs and AuNPs tested by us showed minor differences in inhibition among the bacteria tested. AuNPs inhibited only NASM strains at the same concentration as AgNPs (75 μg/mL). They did not inhibit any *S. aureus* strain. Monitoring the effects of pure AgNPs and AuNPs on biofilm formation revealed significant antibiofilm activity against all tested strains (*S. aureus* and NASM). A greater effect on biofilm formation was observed with AgNPs compared to AuNPs at a concentration of 50 µg/mL. Pure AgNPs and AuNPs did not disrupt staphylococcal biofilms at any tested concentration.

Gugala et al. [45] studied the effectiveness of AgNPs against the growth and biofilm formation of three bacterial strains: *Pseudomonas aeruginosa, S. aureus,* and *Escherichia coli*. Dehkordi et al. [31,46] also observed a strong antibacterial effect against *S. aureus* isolates by determining the MIC with AgNPs at 10 μg/mL. Similarly, low concentrations that inhibit *S. aureus* growth were reported by Elbehiry et al. [31] (12.5 μg/mL). Sevinc-Sasmaz et al. [47] reported the effect of a higher AgNPs concentration (75 μg/mL) on *S. aureus*. These results align with our current findings on the antibacterial effect of AgNPs against staphylococci.

In the study by Penders et al. [48], differences are evident. In their study, they did not observe any antibacterial effect even at high AuNP concentrations (250 or 500 mg/mL), although their main goal was to evaluate biological activity based on the different shapes of the prepared NPs. Nonetheless, other studies have also demonstrated the antibacterial and antibiofilm effects of AuNPs [49,50,51]. The authors of the individual works unanimously confirmed strong biological activity. Hernández-Sierra et al. also reported a strong effect of AuNPs at 197 μg/mL against *Streptococcus mutans*. Overall, many published studies show high variability, not only in results but also in methodology and interpretation [52].

The advanced procedure of NPs preparation is a biosynthesis method that utilizes natural resources such as plant extracts, microorganisms, and their products, as well as biopolymers. Biosynthesized NPs are more biocompatible than chemically synthesized NPs and demonstrate lower cytotoxicity, making them suitable for biomedical applications. These methods allow precise control of reaction parameters to regulate size, shape, and surface chemistry, enabling customization for specific applications [44,53].

Biosynthesized AgNPs-AV and AuNPs-AV were produced in our study using an extract from *Alchemilla vulgaris*, which acted as both a reducing and stabilizing agent. Our results show that the strong antibacterial and antibiofilm properties are due to the biosynthesized AgNPs-Av and AuNPs-Av. Notably, these NPs inhibited bacterial growth at 50 μg/mL, while significant suppression of biofilm formation was observed at just 25 μg/mL.

Our findings agree with previous reports, which also show significant antibacterial and antibiofilm effects of biosynthesized AgNPs and AuNPs. In particular, Chegini et al. described effective activity against MRSA [44], using AgNPs stabilized with C-phycocyanin. Using 7.4 μg/mL NPs, we effectively inhibit bacterial growth and prevent biofilm formation. Mohammed et al. [54] observed strong antibacterial activity of licorice-derived AgNPs against MRSA. Similar results to ours were also reported by Hamid et al. [55], who noted strong antibiofilm activity of the plant extract *Carthamus tinctorius*. Swolana and Wojtyczka [24] focused on the application of biosynthesized NPs against various bacterial species, including staphylococci. Based on the published results, the MIC of the tested NPs consistently falls between 0.19 µg/mL and 250 µg/mL. This indicates that incorporating various natural bioactive compounds into NPs preparations demonstrates their wide-ranging potential for targeted therapy. Oh et al. [56] found enhanced antibacterial effects of AuNPs when using seaweed extracts containing phlorotannins. Specifically, biosynthesizing AuNPs with the ethyl acetate fraction of *Eisenia bicyclis* resulted in 44.64% inhibition against *S. aureus* at 128 µg/mL.

Our research showed that neither AuNPs-Av nor AuNPs disrupts the bacterial biofilm layer. However, AgNPs-Av clearly eradicated the biofilm at a concentration of 50 μg/mL. So far, few studies have been published on the disruption of bacterial biofilms by NPs to thoroughly evaluate their effectiveness in eradication. A recent study by Takahashi and Moriguchi [57] is relevant to our research because it demonstrated disruption of the biofilm formed by the bacterial strain *S. epidermidis*. Hosnedlova et al. [58] concluded that NPs disrupted the biofilm, with their study showing the eradication of S. aureus biofilm by various NPs at levels ranging from 42 to 63%.

NPs can exert antibacterial effects and synergize with ATB, allowing for reduced antibiotic doses to subinhibitory levels [28]. The advantage of the synergistic effect between antibiotics and NPs is the potential to lower antibiotic dosages to subinhibitory levels. This reduces their toxicity to the organism while keeping the same inhibitory activity without affecting bacteria at subinhibitory levels. The synergy can broaden the range of antibiotics that work against bacteria, prevent antibiotics from being broken down too quickly, and enhance their buildup at the infection site. Lastly, it can restore the ability of resistant bacterial strains to respond to antibiotics.

Ampicillin remains recommended in guidelines for empirical treatment of methicillin-susceptible *S. aureus* (MSSA) [40]. Therefore, it was the ideal candidate to observe the synergistic effect of subinhibitory concentrations with NPs. Both AgNPs and AuNPs are toxic to cells at high concentrations; therefore, it is important to use the lowest effective drug concentrations in therapy, as demonstrated by this study. Our research indicates that both AgNPs-Av and AuNPs-Av significantly inhibit bacterial growth at subinhibitory concentrations (25 μg/mL) when used together with subinhibitory ampicillin. Both NPs also significantly inhibited biofilm formation at 12.5 μg/mL, half the antibacterial testing concentration, when combined with subinhibitory ampicillin for each strain. The most significant differences were seen in the eradication effects. Differences emerged not only between AgNPs and AuNPs but also within each type across strains. The subinhibitory AgNPs-Av concentration (25 μg/mL) with ampicillin eradicated biofilm in most strains, except MRSA and multiresistant *S. pseudintermedius*. Notably, the synergistic effect of AuNPs-Av (25 μg/mL) with ampicillin was surprising. This combination eradicated MRSA biofilms and all NASM strains, but did not eliminate *S. aureus* biofilms.

Similarly, Surwade et al. [59] chose ampicillin to investigate the synergistic effect of antibiotics with NPs. By examining the synergistic activity of AgNPs with ampicillin, they observed greater effectiveness at lower ampicillin concentrations, contrary to the expected increase at higher concentrations. They also demonstrated that ampicillin had strong antibacterial effects when combined with AgNPs, even at 0.03 µg/mL. NPs form a complex with ampicillin, which disrupts the peptidoglycan of the cell wall and, because of their positive charge, also interacts with negatively charged transmembrane proteins. This interaction may cause cell membrane breakdown and block transport channels. In contrast, Murei et al. observed minimal inhibitory activity against *S. aureus* using plant extracts conjugated to ampicillin and AgNPs, which led to a significantly lower MIC value (0.05 mg/mL) than with ampicillin alone [60]. Awad et al. [61] demonstrated that combining vancomycin with AgNPs enhances its effectiveness at lower concentrations. Similarly, Masadeh et al. [62] found that the synergistic effect of NPs with an antibiotic not only produced antibacterial effects but also inhibited biofilm formation and eradicated biofilms. They reported MICs against bacteria ranging from 0.0625 to 0.125 mg/mL and biofilm-eradicating concentrations from 0.125 to 0.25 mg/mL. They also discovered that combining AgNPs with antibiotics lowered the MIC to below 0.00195 mg/mL.

Our findings emphasize the importance of rational combination therapy to improve antibiotic effectiveness and lower bacterial resistance.

## 4. Materials and Methods

### 4.1. Selection of Bacterial Strains for Testing

Two reference strains of *S. aureus* from the Czech Collection of Microorganisms (Brno, Czech Republic) were selected to evaluate the antibacterial, antibiofilm, and biofilm-eradication effects of NPs. It was *S. aureus* CCM 4750, which was resistant to methicillin, and *S. aureus* CCM 4223 produced biofilm. Four additional strains tested included Non-*aureus* staphylococci and mammaliicocci (NASM) clinical isolates with multidrug resistance: *S. xylosus* and *Mammaliicoccus sciuri* (originally *S. sciuri*), both from cows with subclinical mastitis, and *S. equorum* and *S. pseudintermedius* from dog skin [63].

The minimal inhibitory concentration (MIC) of antibiotics of the tested strains was determined using a colorimetric microdilution method with automatic reading on the Miditech system (Bratislava, Slovakia) [64]. Building on this, the Miditech software version Expert 09/2024 (Bel-MIDITECH sro, Bratislava, Slovakia; cat. no. 002002) assessed the minimum inhibitory concentration for 20 antibiotics and identified resistance mechanisms based on the MIC breakpoints according to the EUCAST 2024 guidelines (version 14.0) (CLSI. Part B. CLSI) [65] vs. FDA Breakpoints (CLSI M100-Ed33) Version 1.0; Clinical & Laboratory Standards Institute (CLSI): Wayne, PA, USA, 2023).

Clinical isolates were confirmed to the species level through 16S rRNA gene sequencing. The resulting sequences were uploaded to a database, and each isolate was assigned an accession number (Table A1). The system then classified isolates as susceptible or resistant. Furthermore, all bacterial strains tested in this study demonstrated biofilm-forming ability.

**Figure A1:**
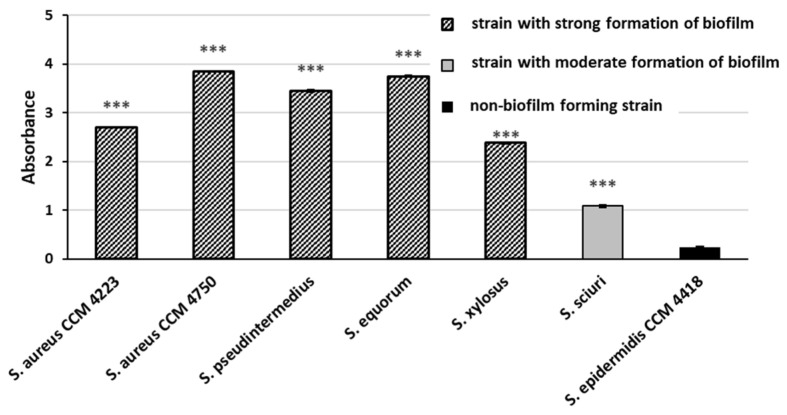
Biofilm formation in 6 tested isolates. Description: *** significant production of biofilm *p* < 0.001; CCM 4223 *S. aureus*—positive control, biofilm-forming strain; CCM 4418 *S. epidermidis*—negative control, non-biofilm-forming strain.

The biofilm-forming ability and rate of the tested strains (Figure A1) were evaluated using a modified colorimetric method based on crystal violet staining, as described by O’Toole et al. [66].

### 4.2. Biosynthesis of Ag and Au Nanoparticles

#### 4.2.1. Extraction Procedure

Dried leaves of *Alchemilla vulgaris* (Juvamed, Rimavská Sobota, Slovakia) were milled into powder form. The extract was prepared using 5 g of drug suspended in 100 mL of extraction solvent (ethanol). The suspension was stirred at room temperature for 2 h. After the filtration, the extract was immediately used for NPs preparation.

#### 4.2.2. Biosynthesis

As the precursor for AgNPs and AuNPs synthesis, a water solution of silver nitrate (AgNO_3_) (Mikrochem, Pezinok, Slovakia), chloroauric acid (HAuCl_4_·3H_2_O) (Sigma Aldrich, St. Louis, MO, USA), respectively, was used with concentrations of 300 μg/mL. The solutions of precursors were kept in the dark at room temperature for further use.

The solutions of precursors were added to the extract in a 9:1 ratio, and these mixtures were stirred at 80 °C until the reactions were completed. The reaction process of NPs preparation was detected visually by color change and by UV/Vis spectra monitoring in the range 750–350 nm of wavelength to obtain surface plasmon resonance bands. The UV-Vis spectra were measured by a UV-Vis spectrophotometer Cary 60 (Agilent Technologies, Santa Clara, CA, USA). The single gold and silver NPs were obtained by washing the NPs multiple times, and used for antibacterial effect studies as Ag/AuNPs. The Ag/AuNPs-Av are NPs prepared without washing and were used as nanosuspensions. The prepared NPs were directly used for antibacterial studies.

#### 4.2.3. Characterization of Nanoparticles

Size distribution measurements of the obtained nanosuspensions were performed using the laser diffraction analysis methodology by the Mastersizer 3000 particle size analyzer (Malvern Panalytical, Westborough, MA, USA). The refractive index was 1.330 for AgNPS, and 1.335 for AuNPS, and measurements were repeated three times for all the samples.

#### 4.2.4. FE-SEM Measurements

The morphologies of prepared AuNPs and Ag NPs were also characterized by field-emission scanning electron microscopy (LEOL JSM-IT700HR, Tokyo, Japan) with an accelerating voltage of 15 kV. A sample of NPs for FE-SEM observation was prepared on carbon-coated copper grids.

### 4.3. Statistical Analysis

To assess antibacterial, antibiofilm, and biofilm-eradicating activities, statistical analysis was performed using one-way ANOVA with Dunnett’s test in Prism 8.3.0 software. Specifically, MIC was defined as the lowest dilution at which no visible growth of the microorganism occurred (i.e., no turbidity). Furthermore, all testing was performed in triplicate, and the mean value ± SD was calculated for each isolate.

### 4.4. Preparation of Bacterial Strains for Testing

Bacterial strains for NPs testing were grown for 24 h at 37 °C on blood agar. After incubation, single colonies were suspended in saline to achieve a 1 McFarland standard of turbidity.

### 4.5. Antibacterial Effect of Nanoparticles and Nanoparticles with Antibiotics

The antibacterial effects of AgNPs and AuNPs and their synergistic interactions with antibiotics were assessed by determining minimum inhibitory concentrations (MICs). Testing followed guidelines from the European Committee on Antimicrobial Susceptibility Testing (EUCAST) and the Clinical Laboratory Standards Institute (CLSI) (EUCAST 2003, CLSI 2006) [65]. We evaluated the antibacterial activity of NPs, both alone and in combination with antibiotics, by diluting them into nutrient broth. Diluted samples were incubated with bacterial strains in a 96-well microtiter plate (Brand, Wertheim, Germany). Different NPs concentrations, from 50 to 125 μg/mL, were prepared from a 300 μg/mL stock and diluted in modified BHI (mBHI—Brain Heart Infusion Broth, HiMedia Laboratories, Mumbai, India) containing 1% glucose and 2% NaCl. Various subinhibitory concentrations of antibiotic, ranging from 0.125 to 32 μg/mL, were prepared from the stock solution (10 mg/mL) along with subinhibitory concentrations of NPs. NPs and their combinations with antibiotics in mBHI were mixed with 100 μL of bacterial strains (1 McFarland standard) in the wells. Each dilution was tested in triplicate. The tested concentrations of NPs alone were 125, 100, 75, 50, and 25 μg/mL. For synergistic effect assessment, the NPs concentration was fixed at 25 μg/mL. The antibiotic MIC and two two-fold dilutions were determined for each bacterial strain. Antibiotic concentrations varied depending on MIC and resistance levels, as listed in Table A1. Table 1 presents the subinhibitory concentrations used in synergistic testing of antibiotics with NPs.

The final bacterial density in both wells containing NPs (with and without antibiotic) was 0.5 McFarland. This corresponds to 1.5 × 10^8^ CFU/mL. Control wells contained either broth with NPs or NPs with an antibiotic, saline as a background. Pure broth and saline with the bacterial strain served as positive controls for bacterial growth. Additionally, pure broth with saline (without bacterial culture) was prepared as a negative control.

In the other wells, test solutions and broth containing NPs, as well as NPs with an antibiotic, were pipetted. After 24 h of incubation at 37 °C with constant shaking, growth inhibition was measured spectrophotometrically by recording absorbance at 600 nm using a Synergy reader 4 (Merck, Darmstadt, Germany).

### 4.6. Antibiofilm Effect of Nanoparticles and Nanoparticles Combined with Antibiotics

Biofilm inhibition by NPs and their combinations with antibiotics was tested in microtiter plates with 100 μL of bacterial suspension (1 McFarland) and 100 μL of mBHI, containing the chosen concentrations of NPs and NPs with antibiotics. These concentrations aligned with those in the bacterial growth inhibition test and were assessed in triplicate. A bacterial suspension without NPs served as the biofilm-positive control, broth alone as the negative control, and pure NPs without bacteria were used for background subtraction. Plates were incubated at 37 °C for 24 h. Biofilm activity was then assessed using a modified O’Toole protocol. Inhibition was measured spectrophotometrically at 550 nm with a Synergy reader 4 (Merck, Darmstadt, Germany).

### 4.7. Biofilm-Eradication Effect of Nanoparticles and Nanoparticles with Antibiotics

The effect of NPs and NPs with antibiotics on biofilm eradication was monitored at the same selected concentrations as in the change-of-experiments in this study. Bacterial strain (100 μL, 1 McFarland) and 100 μL mBHI (containing the chosen concentrations of NPs and NPs with antibiotics) were mixed in microtiter plate wells and incubated for 24 h at 37 °C to form a continuous biofilm. mBHI was then replaced with fresh mBHI medium containing the test substances. Plates were incubated again at 37 °C for 24 h. After incubation, biofilm was evaluated with a modified O’Toole protocol [66]. Biofilm presence was assessed by measuring absorbance at 550 nm using a Synergy reader 4 (Merck, Darmstadt, Germany).

## 5. Conclusions

In this work, the biological activity of AgNPs and AuNPs was monitored and compared to biosynthesized NPs prepared using *Alchemilla vulgaris* extract (AgNPs-Av and AuNPs-Av). Based on the results, it can be concluded that the prepared AgNPs-Av and AuNPs-Av had average sizes of 71.9 nm and 23.1 nm, respectively. The plant extract reduced the amount of silver and served as a stabilizing and capping agent. Our study indicates a strong inhibitory activity of biosynthesized NPs against the growth of planktonic cells and the formation of staphylococcal biofilms. Moreover, AgNPs-Av eradicated the pre-existing biofilm. The most significant results were obtained by testing the interaction between NPs and ampicillin at subinhibitory concentrations. Reduced concentrations of NPs significantly potentiated the effect of ampicillin, even at its concentrations many times lower than the MIC against the tested staphylococcal strains. Therefore, this study demonstrated the potential of using NPs in combination with low concentrations of antibiotics in human and veterinary medicine.

## Figures and Tables

**Table 1:** Subinhibitory concentrations of synergistic testing of ATB with NPs.

Tested Isolates	1. Concentration	2. Concentration	3. Concentration
*S. aureus* CCM 4750	32 mg/L	16 mg/L	8 mg/L
*S. aureus* CCM 4223	0.5 mg/L	0.25 mg/L	0.125 mg/L
*S. pseudintermedius*	2 mg/L	1 mg/L	0.5 mg/L
*S. equorum*	0.5 mg/L	0.25 mg/L	0.125 mg/L
*S. xylosus*	32 mg/L	16 mg/L	8 mg/L
*M. sciuri*	16 mg/L	8 mg/L	4 mg/L

## Data Availability

The original contributions presented in this study are included in the article. Further inquiries can be directed to the corresponding author.

## Abbreviations

The following abbreviations are used in this manuscript:

PH(2″)-AC(6′) type	aminoglycoside resistance
AMP	ampicillin
CIP	ciprofloxacin
CLI	clindamycin
COT	trimethoprim + sulfonamide
DNA	deoxyribonucleic acid
ERY	erythromycin
EUCAST	European Committee on Antimicrobial Susceptibility Testing
FOX	cefoxitin
GEN	gentamicin
CHL	chloramphenicol
LNZ	linezolid
MALDI-TOF MS	matrix-assisted laser desorption ionization–time of flight mass spectrometry
mBHI	modified brain heart infusion broth
MFX	moxifloxacin
MIC	minimum inhibitory concentration
MLSB_c_	Constitutive resistance to macrolide-lincosamide-streptogramin B
MLSB_i_	Inducible resistance to macrolide-lincosamide-streptogramin B
MRCoNS	methicillin-resistant coagulase-negative staphylococci
MSCRAMM	microbial surface components recognizing adhesive matrix molecules
MDR	multidrug resistance
NIT	nitrofurantoin
NPs	nanoparticles
OXA	oxacillin
PCR	polymerase chain reaction
PIA	polysaccharide intercellular antigen
RIF	rifampicin
SAM	ampicillin + sulbactam
TEC	teicoplanin
TET	tetracycline
TGC	tigecycline
TMP	trimethoprim
TZP	piperacillin + tazobactam
VAN	vancomycin

## Appendix A

**Table A1:** Minimum inhibitory concentration and mechanism of resistance in tested Staphylococci.

	*S. aureus* CCM 4223	*S. aureus* CCM 4750	*S. pseudintermedius*	*S.equorum*	*S. xylosus*	*S. sciuri*
Accession number			PV688034	PV688033	PX904986	PX904985
Antimicrobial resistance						
AMP	1	64	4	1	64	32
SAM	0.25	16	0.5	1	4	4
TZP	0.5	128	0.5	4	1	4
OXA	0.5	8	0.25	2	8	8
FOX	2	32	0.5	4	32	32
GEN	0.5	128	0.5	0.25	0.25	0.5
CIP	0.5	8	4	0.25	0.5	0.125
MFX	0.0625	2	2	0.125	0.125	0.0313
ERY	0.5	16	16	2	1	0.0625
CLI	0.0625	8	8	0.25	0.25	0.25
LNZ	4	2	2	1	1	0.5
RIF	0.0313	8	0.0313	0.0313	0.25	0.0625
VAN	1	2	1	1	1	0.25
TEC	1	2	0.25	0.125	0.5	0.125
TET	0.5	32	32	32	1	0.25
TGC	0.0313	0.0625	0.0625	0.0313	0.0625	0.0625
CHL	8	8	32	2	2	0.5
TMP	4	0.25	2	1	32	32
COT	0.5	0.5	2	0.25	1	2
NIT	8	8	8	1	8	1
Mechanisms of resistance						
Resistance to penicillins	+	-	-	-	-	-
mecA	-	-	-	-	-	-
MRSA	-	+	-	-	-	-
MRCoNS	-	-	-	+	+	+
PH(2″)-AC(6′)	-	+	-	-	-	-
MLSB/c	-	+	+	-	-	-
MLSB/i	-	-	-	-	-	-
Multiresistance	-	+	+	+	-	-
Biofilm formation	Strong	Strong	Strong	Strong	Strong	Moderate

Description: AMP = ampicillin, SAM = ampicillin + sulbactam, TZP = piperacillin + tazobactam, OXA = oxacillin, FOX = cefoxitin, GEN = gentamicin, CIP = ciprofloxacin, MFX = moxifloxacin, ERY = erythromycin, CLI = clindamycin, LNZ = linezolid, RIF = rifampicin, VAN = vancomycin, TEC = teicoplanin, TET = tetracycline, TGC = tigecycline, CHL = chloramphenicol, TMP = trimethoprim, COT = trimethoprim + sulfonamide, NIT = nitrofurantoin, MLSB/c—constitutive MLSB, MLSB/i—inducible MLSB; PH(2″)-AC(6′)—aminoglycozide resistance type PH(2″)-AC(6′). Values highlighted in green represent resistance to the given antimicrobial agents.

**Table A2:** Comparison of the significance of the lowest effective concentrations of NPs in the tested staphylococcal isolates.

	Washed NPs		Biosynthesized NPs	
	AgNPs	AuNPs	AgNPs-Av	AuNPs-Av
Antibacterial effect of NPs against Staphylococci				
*S. aureus* CCM 4750	75 µg/mL	-	50 µg/mL	50 µg/mL
*S. aureus* CCM 4223	75 µg/mL	-	50 µg/mL	50 µg/mL
*S. equorum*	75 µg/mL	75 µg/mL	50 µg/mL	50 µg/mL
*S. pseudintermedius*	75 µg/mL	75 µg/mL	50 µg/mL	50 µg/mL
*S. xylosus*	75 µg/mL	75 µg/mL	50 µg/mL	50 µg/mL
*S. sciuri*	75 µg/mL	100 µg/mL	50 µg/mL	50 µg/mL
Antibiofilm effect of NPs against Staphylococci				
*S. aureus* CCM 4750	50 µg/mL	75 µg/mL	25 µg/mL	50 µg/mL
*S. aureus* CCM 4223	50 µg/mL	50 µg/mL	25 µg/mL	25 µg/mL
*S. equorum*	50 µg/mL	50 µg/mL	25 µg/mL	25 µg/mL
*S. pseudintermedius*	50 µg/mL	75 µg/mL	25 µg/mL	50 µg/mL
*S. xylosus*	50 µg/mL	50 µg/mL	25 µg/mL	25 µg/mL
*S. sciuri*	50 µg/mL	50 µg/mL	25 µg/mL	25 µg/mL
Biofilm-eradication effect of NPs against Staphylococci				
*S. aureus* CCM 4750	-	-	50 µg/mL	-
*S. aureus* CCM 4223	-	-	50 µg/mL	-
*S. equorum*	-	-	50 µg/mL	-
*S. pseudintermedius*	-	-	50 µg/mL	-
*S. xylosus*	-	-	50 µg/mL	-
*S. sciuri*	-	-	50 µg/mL	-
